# Ultrafast control of third-order optical nonlinearities in fishnet metamaterials

**DOI:** 10.1038/srep28440

**Published:** 2016-06-23

**Authors:** Alexander S. Shorokhov, Kirill I. Okhlopkov, Jörg Reinhold, Christian Helgert, Maxim R. Shcherbakov, Thomas Pertsch, Andrey A. Fedyanin

**Affiliations:** 1 Lomonosov Moscow State University, Faculty of Physics, Moscow, 119991, Russia; 2 Friedrich Schiller University Jena, Institute of Applied Physics, Abbe Center of Photonics, Jena, 07743, Germany

## Abstract

Nonlinear photonic nanostructures that allow efficient all-optical switching are considered to be a prospective platform for novel building blocks in photonics. We performed time-resolved measurements of the photoinduced transient third-order nonlinear optical response of a fishnet metamaterial. The mutual influence of two non-collinear pulses exciting the magnetic resonance of the metamaterial was probed by detecting the third-harmonic radiation as a function of the time delay between pulses. Subpicosecond-scale dynamics of the metamaterial’s *χ*^(3)^ was observed; the all-optical *χ*^(3)^ modulation depth was found to be approximately 70% at a pump fluence of only 20 *μ*J/cm^2^.

Artificially nanostructured materials with novel electromagnetic properties, currently referred to as metamaterials, were originally introduced to enable the on-demand tailoring of their constitutive parameters. Negative refractive indices^1^,^2^, epsilon-near-zero phenomena^3^, form birefringence^4^, chirality^5^,^6^, and other properties of metamaterials have been reported. Because the constitutive relations for these materials consist of both linear and nonlinear terms, enabling artificial control over electromagnetic nonlinearities is a very feasible task. Nonlinear metamaterials have constituted their own research field for over a decade^7^, and experimental results vary, for example, from self-action nonlinearities in varactor-loaded magnetic metamaterials^8^,^9^ and magnetoelastic self-action^10^ to inverse phase-matching^11^ and light-controlled metamaterials^12^, together with novel low-loss all-dielectric nonlinear metamaterials^13^,^14^,^15^,^16^.

In contrast to the case of macroscopic microwave and terahertz metamaterials, the natural obstacles imposed by nanofabrication limitations impede the possibility of scaling nonlinear metamaterials to the optical regime. The development of new nonlinear media for optical radiation is therefore crucial for applications in all-optical switching, and metallic metamaterials may represent a potential source of large plasmonic nonlinearities. Following the basic concepts of local-field-enhanced metal nonlinearities^17^,^18^, plasmon-enhanced harmonic generation and wave mixing^19^,^20^,^21^,^22^ have been observed in metamaterials and plasmonic nanosystems. Most importantly, the symmetry of nanostructured metamaterial may give rise to new nonlinear contributions because of its specific geometry^23^,^24^ or the specific symmetry of the currents of magnetic resonances^25^,^26^,^27^,^28^.

The use of optical metamaterials and plasmonic nanosystems for all-optical switching has been extensively discussed^27^,^29^. The photoinduced modulation of linear dielectric constants in metamaterials via free-carrier generation in silicon^30^, graphene^31^ or aluminum^29^; the reorientation of liquid crystals^32^; and coherent control^33^,^34^ have been demonstrated. Notably, the shortest switching times have been achieved by utilising ultrafast free-electron gas heating and thermalization in plain metallic films^35^,^36^, nanoparticles^37^, and nanorod metamaterials^27^; one drawback of these mechanisms lies in the necessity of high-power laser pumping with regenerative-amplifier-scale intensities. Nevertheless, nonlinear susceptibilities are well known to be more highly sensitive to external stimuli than are linear susceptibilities, and femtosecond control over the optical nonlinearities in metamaterials has not been reported to date.

In this contribution, we report an experimental demonstration of the ultrafast modulation of the third-order optical nonlinearities of a fishnet metamaterial excited at its magnetic resonance by femtosecond laser pulses. By observing the third-harmonic radiation generated as a function of the time delay between two consecutive pulses, we probed the photoinduced third-order nonlinear susceptibility dynamics. The experimental results reveal *χ*^(3)^ modulation on the subpicosecond time scale and relaxation times with a modulation depth of 70% under a very modest pump peak intensity of 90 MW/cm^2^ and a fluence of 20 *μ*J/cm^2^. The underlying microscopic picture of nonlinear control based on free-electron gas heating and thermalization explains the ultrashort *χ*^(3)^ relaxation times.

## Results and Discussion

**Figure 1:**
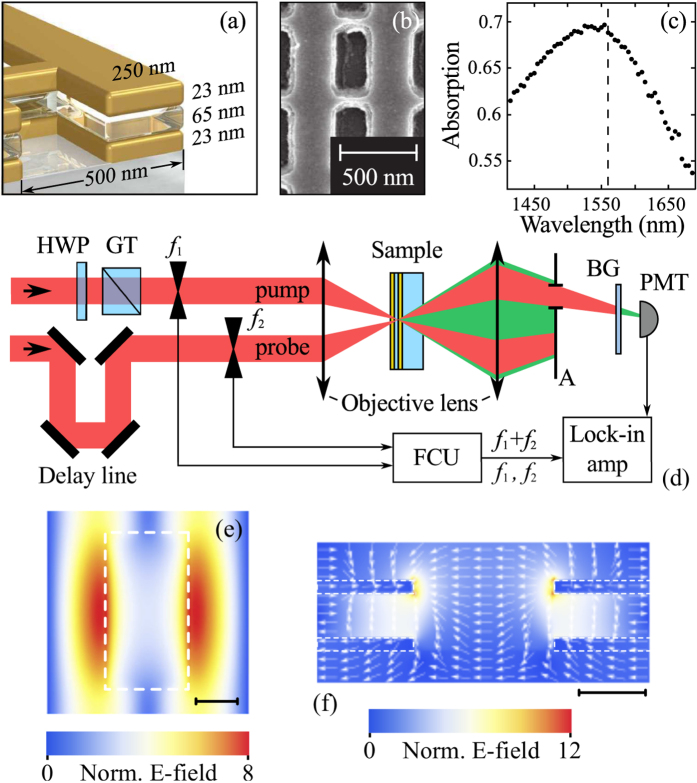
(**a**) Schematic illustration of the studied fishnet metamaterial. (**b**) Scanning electron micrograph of the sample. (**c**) Absorption spectrum of the sample acquired at 10° incidence. The dashed line indicates the carrier wavelength of the pump laser. (**d**) Schematic illustration of the experimental setup: HWP represents a half-wave plate, GT represents a laser-grade Glan-Taylor polarizer, the beams were chopped at frequencies of *f*_1_ and *f*_2_, BG indicates a 4 mm thick Schott BG39 filter glass, PMT represents a photomultiplier tube, FCU represents a frequency-conditioning unit, and A represents an iris aperture. (**e**) Calculated at 1550 nm *E*-field distribution plotted in the horizontal plane sliced through the center of the MgO slab. White dashed line represents the borders of the hole perforated through the structure. (**f**) Calculated at 1550 nm *E*-field distribution superimposed with the normalized field vectors plotted in the vertical plane sliced through the center of the unit cell depicted in (**e**). For both (**e,f**), scale bars denote 100 nm.

A fishnet metamaterial of the same type studied in ref. 25 was used to demonstrate the all-optical control of its third-order optical nonlinearities. The sample consisted of a three-layer Au/MgO/Au heterostructure patterned with an array of rectangular holes and situated on a fused silica substrate. The dimensions, a scanning electron micrograph and the absorption spectrum of the sample are shown in Fig. 1(a–c), respectively. Careful experimental and numerical analyses revealed a magnetic resonance at a wavelength of 1.55 *μ*m and polarization along the lesser of the hole sides. This resonance sustains the antisymmetric movement of free-electron currents in the two gold layers and causes the oscillation of a nonzero magnetic moment at the external electromagnetic field frequency. Calculated *E*-field profiles excited inside the structure at 1550 nm are provided in Fig. 1(e–f) and clearly illustrate this statement. Electric fields are in the reversed phase in top and bottom layers of the fishnet metamaterial (Fig. 1(f)) forming two plasmon waves propagating oppositely along top and bottom interfaces between gold and MgO layers. It leads to the formation of standing waves in the dielectric slab of the structure (Fig. 1(e)). Based on the spectral full width at half maximum of the resonance, the lifetime of the mode can be estimated to be approximately 50 fs.

Local field enhancement has previously been observed in fishnet metamaterials— see, e.g. ref. 38—resulting in enhanced nonlinearities. The third-order optical nonlinearities are of particular interest because of the large reported *χ*^(3)^ values of bulk gold^39^ and the retardation peculiarities observed in metamaterials^25^. To probe the photoinduced modulation of *χ*^(3)^, a setup based on an Er^3+^-doped fiber femtosecond laser was built to observe time-dependent third-harmonic generation (THG). The basic design of the setup is depicted in Fig. 1(d). A train of 220 fs pulses with a central wavelength of *λ* = 1560 nm was split into two beams with an approximately equal power; the first and second beams were focused by an objective lens with a numerical aperture of *NA* = 0.5 and a working distance of *WD* = 14 mm onto the sample surface at a mutual angle of approximately 20° after passing through a polarization conditioner and a delay line, respectively. The horizontally polarized beams, corresponding to the resonant excitation of the sample, were brought together to a waist with a diameter of 25 *μ*m, leading to a maximum single-beam peak intensity of 90 MW/cm^2^ and a fluence of 20 *μ*J/cm^2^ in the plane of the sample. Based on the reproducibility of the THG values obtained from the same sample area at the same pump powers, it was concluded that the sample suffered no irreversible damage. A double-frequency 1:1 optical chopper was installed before the objective lens to chop the first and second beams at frequencies of *f*_1_ = 500 Hz and *f*_2_ = 600 Hz, respectively. This enabled the measurement of both THG contributions originating from each beam independently, locked in at either *f*_1_ or *f*_2_, and the THG resulting from the interaction of the beams, locked in at a frequency of *f*_1_ + *f*_2_. The use of two types of long-pass color filters revealed that the detected signal arose from light with a spectrum lying between 510 nm and 530 nm. This range includes the THG wavelength of *λ* = 520 nm, demonstrating that the detected signal can be attributed to the THG and not to any other source, e.g., three-photon luminescence.

**Figure 2:**
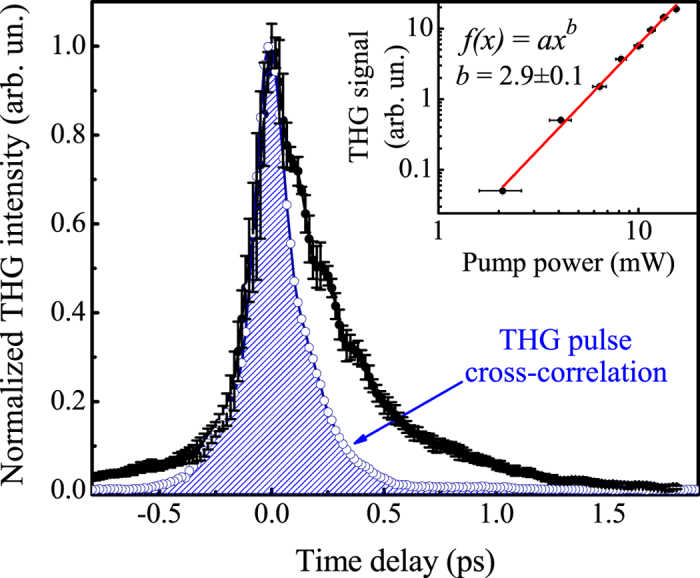
Filled black dots: THG of the sample as a function of the time delay between pulses, as detected at the *f*_1_ + *f*_2_ frequency of the chopper. Blue solid curve and dots: THG of the silica substrate as a function of the time delay between pulses, as detected at *f*_1_ + *f*_2_. Inset: THG signal of the sample measured at *f*_1_ when the second beam was blocked as a function of the power of the first beam. The solid red line represents a fit to the power function *f*(*x*) = *ax*^*b*^, indicating a power of *b* = 2.9 ± 0.1.

The THG from either of the beams was measured by analyzing the PMT signal at the frequencies of *f*_1_ and *f*_2_ separately, with the aperture removed. Blocking one of the beams yielded the same THG signal from the other. As expected, a cubic (curved slope equal to 2.9 ± 0.1) dependence of the single-beam THG intensity on the pump power was observed (see the inset of Fig. 2).

However, a complicated picture of the mutual modulation of the THG signal was observed upon bringing the two beams together at the sample site. The normalized time-resolved THG dependence measured by locking in the PMT signal at *f*_1_ + *f*_2_ is shown by the filled dots in Fig. 2. Here, the abscissa values indicate the time delay between pulses. Superimposed on the sample’s THG dependence is the normalized THG dependence measured in the silica substrate. The maximum THG signal from the sample measured at the lock-in reference frequency of *f*_1_ + *f*_2_ was approximately 10 times higher than the signal from the substrate and approximately 3 times higher than the THG signal from the sample measured at *f*_1_ with the second beam blocked. On the one hand, the THG of the sample at close-to-zero time delay values resembled the shape of the cross-correlation function, indicating a coherent process of THG enhancement. On the other hand, there existed time delay values, e.g., from 0.5 ps to 1.5 ps, at which the cross-correlation function was nearly zero and the pump beam was seen to exert a significant influence on the THG from the probe beam. The observed effect can be explained in terms of modification of the electron plasma nonlinear susceptibility under the strong impact of the pump laser beam with the following relaxation of the electronic subsystem through the interaction with the lattice. Such processes will be considered in more details further in the manuscript.

**Figure 3:**
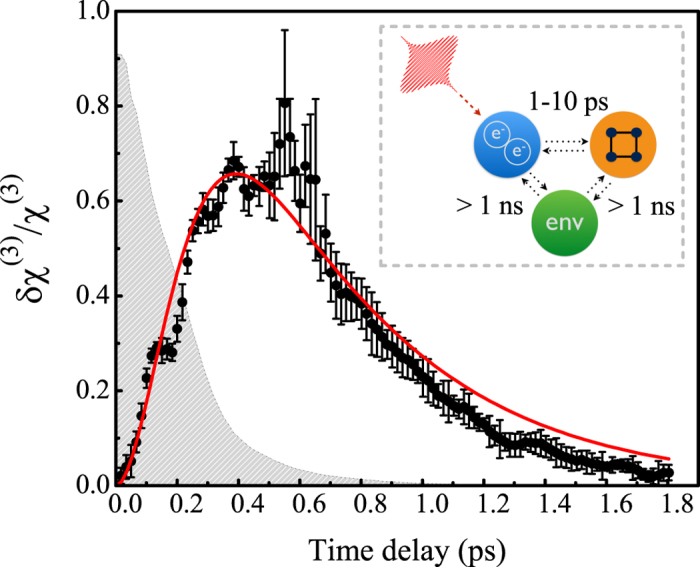
The relative change in the effective third-order nonlinear susceptibility of the fishnet metamaterial as a function of the time delay between the pump and probe pulses (black dots) and its fit to Eq. (7) **(red curve).** The gray dashed area represents the normalized cross-correlation function of the laser pulses. Inset: illustration of the photoinduced relaxation processes occurring in the structure.

When a powerful laser pulse impinges on a gold film, it modifies the linear dielectric constants of the medium, as many authors have previously shown^35^,^36^. This implies that in a pump-probe experiment, when the stronger of the two pulses drives the (electronic) system out of its thermal equilibrium state and the weaker of the two probes this transient state, the weaker pulse is modulated by the stronger one. The specific form of the temporal dependence of the dielectric permittivity, *ε*(*t*), is defined by the microscopic dynamics of the electron gas evolution under laser pulse stimulation. In the case of gold and the pump photon energy used in this study, the following processes affect the linear optical properties of the sample: (a) the intraband photoexcitation of electrons and the formation of a non-equilibrium electron distribution, (b) electron thermalization and the establishment of a new electron gas temperature, (c) electron-phonon relaxation, and (d) energy transfer from the gold to the environment; see the inset of Fig. 3. Process (a) has been shown to occur in the sub-100 fs regime^37^; hence, it is regarded as instantaneous with respect to the pulse length used in this paper. Process (d) is slow, with a relaxation time of hundreds of picoseconds. This process is minimally important with regard to the present research because both the linear and nonlinear susceptibilities of noble metals can be reasonably described based on the properties of the electronic subsystem^40^. Finally, the characteristic relaxation times of processes (b) and (c) are in the subpicosecond range, and thus, these processes explain the observed THG dynamics.

The effects on the electronic subsystem can contribute to the transient nonlinear response of a metamaterial in three different ways: (i) changes in the linear transmittance coefficient at both the fundamental and third-harmonic wavelengths, (ii) modulation of the local field factor of the structure at both the fundamental and third-harmonic wavelengths, and (iii) modification of the effective nonlinear susceptibility of the sample. Using the finite-difference time-domain (FDTD) method, we theoretically and numerically calculated the relative contributions of the terms related to (i) and (ii), which are equal to 10^−3^–10^−4^ and thus cannot explain the observed modulation in the third-harmonic generation signal. Therefore, these contributions will be neglected.

The general expression for nonlinear third-order polarization in the isotropic response approximation can be written as follows^41^:


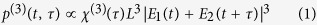


Here, *E*_1_ and *E*_2_ are the electric fields that correspond to the pump and probe pulses, respectively; *τ* is the time delay between them; and *L* is the local field factor at the fundamental frequency. The local field factor of the sample at the third-harmonic frequency is on the order of unity; therefore, we neglect it. Moreover, for simplicity, we will assume that the effective nonlinear susceptibility of the sample, *χ*^(3)^, is a real scalar value. It can be represented as 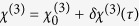, where 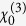 is the non-disturbed static nonlinear susceptibility of the metamaterial and *δχ*^(3)^(*τ*) is the time-dependent contribution. The overall time-averaged THG signal from the sample can then be written as follows:


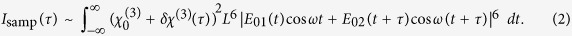


Here, the envelopes 
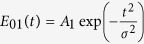
 and 
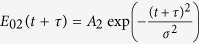
 are defined as Gaussian. After the phase-averaging procedure^42^, we can deduce *δχ*^(3)^(*τ*) as a function of the measured dependences (see the Supplementary Information):


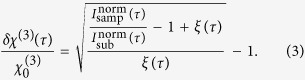


The “norm” superscript denotes the fact that both *I*(*τ*) dependences should be normalized with respect to unity before being substituted into the equation. Here, *ξ*(*τ*) is a function of the pulse parameters:


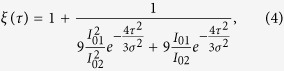


where 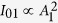 and 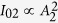; *σ* can be obtained from the normalized cross-correlation function of the pulses. Generally, Eq. (3) is valid for all delay times *τ*. However, in the case of large *τ*, the relative error of the experimental *I*_*sub*_ dependence data is too high to achieve valid results. When *τ* is larger than a couple of *σ*, we can neglect the coherent effects and deduce the relaxation of *δχ*^(3)^(*τ*) by measuring the mixed signal *I*_sam_(*f*_1_ + *f*_2_) and normalizing it with respect to the single-beam signal *I*_sam_(*f*_1_) (see the Supplementary Information):


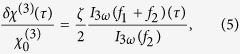


where *ζ* is the proportionality coefficient between the lock-in signal values detected at the frequencies of *f*_2_ and *f*_1_ + *f*_2_; in our case, *ζ* = 2.3. By stitching together Eqs (3) and (5) at *τ* = 800 fs, one can obtain the overall relative photoinduced third-order nonlinear susceptibility dynamics of the sample, as shown in Fig. 3 by means of data points and their corresponding error bars. The dependence corresponds to a standard transient process that involves free-electron plasma; however, this is the first observation of the transient photoinduced dynamics of the *nonlinear* susceptibility of a material.

Here, we quantify the excitation and relaxation times of 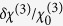. For a weak perturbation of the electron gas, the response function of the system can be presented in the following form^35^:


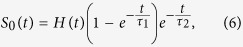


where *H*(*t*) is the step function. The first term introduces a delayed rise of the response function with a time constant *τ*_1_, which characterizes the energy transfer between nonthermal electrons via electron-electron interactions. The exponential decay with a characteristic time of *τ*_2_ describes the relaxation of the excited electrons to the lattice temperature. Because the pulse duration is comparable to the characteristic electron gas relaxation times, the response function must be convolved with the pulse shape. Upon accounting for the finite pulse length, one can finally obtain the following expression for *t* ≥ 0:


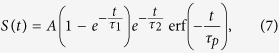


where *τ*_*p*_ is the pulse width, *A* is the normalization factor, and erf(*x*) is the error function. Eq. (7) is in good agreement with the experimental data; the fit parameters are *A* = 2.1 ± 0.4, *τ*_1_ = 280 ± 70 fs, and *τ*_2_ = 500 ± 40 fs, and the pulse width is fixed at *τ*_*p*_ = 220 fs. These values are in good qualitative agreement with those reported previously^35^,^36^, further supporting our initial hypothesis regarding the origin of the observed *χ*^(3)^ modulation.

Figure 3 explicitly illustrates how the nonlinear susceptibility of a fishnet metamaterial evolves over time once free electrons are being excited via intraband transitions. Please note that the relative *χ*^(3)^ modulation reaches 70% at a very modest pump fluence of 20 *μ*J/cm^2^. A similar modulation depth of the linear response of fishnet metamaterials has been achieved at much higher pump levels of approximately 1 mJ/cm^2^ 30,43. Further increase of the modulation can be envisioned in other plasmonic geometries with larger Q’s of the resonance^4^,^44^,^45^. We have, therefore, successfully utilized the fact that the nonlinear response of a medium is much more sensitive than the linear response to the microscopic processes to establish a novel and efficient platform for all-optical switching.

## Conclusions

The subpicosecond all-optical modulation of third-order nonlinearities in a fishnet metamaterial was demonstrated via the third-harmonic generation pump-probe technique. The observed modulation, with a characteristic time scale of less than 1 ps, can be ascribed to the ultrafast relaxation processes occurring inside the metal portions of the structure. The demonstrated nonlinear susceptibility modulation of approximately 70% for low pump fluences of 20 *μ*J/cm^2^ is much higher than that of the linear susceptibility, which could be a useful feature in modern all-optical telecommunication technologies.

## Additional Information

**How to cite this article**: Shorokhov, A. S. *et al*. Ultrafast control of third-order optical nonlinearities in fishnet metamaterials. *Sci. Rep.* **6**, 28440; doi: 10.1038/srep28440 (2016).

## Supplementary Material

Supplementary Information
