# Supplementary material for: Ultrafast control of third-order optical nonlinearities in fishnet metamaterials
Source: Sci Rep. 2016 Jun 23;6:28440. doi: 10.1038/srep28440 (PMC4917860; doi:10.1038/srep28440)
Supplement: Supplementary Information [file srep28440-s1.pdf]

# Ultrafast control of third-order optical nonlinearities in fishnet metamaterials: supporting information

**Alexander S. Shorokhov[1], Kirill I. Okhlopkov[1], Jörg Reinhold[2], Christian Helgert[2], Maxim R. Shcherbakov[1], Thomas Pertsch[2], and Andrey A. Fedyanin[1,*]**

[1]Lomonosov Moscow State University, Faculty of Physics, Moscow, 119991, Russia
[2]Friedrich Schiller University Jena, Institute of Applied Physics, Abbe Center of Photonics, Jena, 07743, Germany
[*]fedyanin@nanolab.phys.msu.ru

## ABSTRACT

Here, we provide the step-by-step evaluation of Eqs. (3) and (5) of the main text. We also provide additional time-resolved experimental data.

The general expression for the nonlinear third-order polarisation under the isotropic response approximation can be written as follows:

$$p^{(3)}(t,\tau) \propto \chi^{(3)}(\tau)L^3\left|E_1(t)+E_2(t+\tau)\right|^3 \tag{S1}$$

Here, $E_1$ and $E_2$ are the electric fields that correspond to the pump and probe pulses, respectively; $\tau$ is the time delay between them; and $L$ is the local field factor at the fundamental frequency. The local field factor of the sample at the third harmonic frequency is on the order of unity; therefore, we neglect it. Moreover, for simplicity, we will assume that the effective nonlinear susceptibility of the sample $\chi^{(3)}$ is a scalar and real value. It can be represented as $\chi^{(3)} = \chi_0^{(3)} + \delta\chi^{(3)}(\tau)$, where $\chi_0^{(3)}$ is the non-disturbed static nonlinear susceptibility of the metamaterial and $\delta\chi^{(3)}(\tau)$ is the time-dependent contribution. The overall time-averaged THG signal from the sample can then be written as follows:

$$I_{\text{samp}}(\tau) \sim \int\limits_{-\infty}^{\infty} (\chi_0^{(3)} + \delta\chi^{(3)}(\tau))^2 L^6 \Big|E_{01}(t)\cos\omega t + E_{02}(t+\tau)\cos\omega(t+\tau)\Big|^6 dt, \tag{S2}$$

Here, the envelopes $E_{01}(t) = A_1 \exp\left(-\frac{t^2}{\sigma^2}\right)$ and $E_{02}(t+\tau) = A_2 \exp\left(-\frac{(t+\tau)^2}{\sigma^2}\right)$ are defined as Gaussian. After the phase averaging procedure, this expands to the following expression:

$$I_{\text{samp}}(\tau) \sim \frac{5}{16}\left(\chi_0^{(3)} + \delta\chi^{(3)}(\tau)\right)^2 L^6 \Big[\int\limits_{-\infty}^{\infty} E_{01}^6(t)dt + 9\int\limits_{-\infty}^{\infty}\left[E_{01}^4(t)E_{02}^2(t+\tau) + E_{01}^2(t)E_{02}^4(t+\tau)\right]dt + \int\limits_{-\infty}^{\infty} E_{02}^6(t)dt\Big], \tag{S3}$$

or, after the evaluation of the integrals:

$$I_{\text{samp}}(\tau) \sim \left(\chi_0^{(3)} + \delta\chi^{(3)}(\tau)\right)^2 L^6 \left[I_{01}^3 + \Psi + I_{02}^3\right], \tag{S4}$$

where

$$\Psi = 9I_{01}^2 I_{02} e^{-\frac{4\tau^2}{3\sigma^2}} + 9I_{01}I_{02}^2 e^{-\frac{4\tau^2}{3\sigma^2}}. \tag{S5}$$

Given that the response of silica is instantaneous, THG signal from the substrate can be presented as:

$$I_{\text{sub}}(\tau) \sim \left(\chi_{sub}^{(3)}\right)^2 L_{sub}^6 \left[I_{01}^3 + \Psi + I_{02}^3\right]. \tag{S6}$$

Let us assume that the intensity of the pump beam is modulated by the square wave function with the $f_1$ frequency and that the intensity of the probe beam is modulated by the square wave function with the $f_2$ frequency. The modification of the nonlinear optical susceptibility $\delta\chi^{(3)}$ is a result of the pump pulse excitation, and thus, it is also modulated at the $f_1$ frequency.

In Eq. (**??**), only $I_1^3$ and $I_2^3$ are modulated at the $f_1$ and $f_2$ frequencies, respectively. All other addends in this sum (see equation (**??**)) are modulated at the $f_1+f_2$ frequency and can be detected by the lock-in amplifier tuned at $f_1+f_2$. Therefore, the detected THG signal from the silica substrate is expressed as follows:

$$I_{\text{sub}}(\tau) \sim \left(\chi_{sub}^{(3)}\right)^2 L_{sub}^6 \Psi. \tag{S7}$$

In the case of the metamaterial, recalling that the nonlinear susceptibility $\delta\chi^{(3)}$ is modulated at the $f_1$ frequency, one can achieve the following expression for the observed THG dependence:

$$I_{\text{samp}}(\tau) \sim \left(\chi_0^{(3)}\right)^2 L^6 \Psi \left[1 + 2\frac{\delta\chi^{(3)}(\tau)}{\chi_0^{(3)}}\xi + \left(\frac{\delta\chi^{(3)}(\tau)}{\chi_0^{(3)}}\right)^2 \xi\right]. \tag{S8}$$

Here, we denote:

$$\xi = 1 + \frac{1}{9\frac{I_{01}^2}{I_{02}^2}e^{-\frac{4\tau^2}{3\sigma^2}} + 9\frac{I_{01}}{I_{02}}e^{-\frac{4\tau^2}{3\sigma^2}}}, \tag{S9}$$

where $\sigma$ can be obtained from the normalized cross-correlation function of the pulses. From Eqs. (**??**) and (**??**), one can obtain:

$$\frac{I_{\text{samp}}^{\text{norm}}}{I_{\text{sub}}^{\text{norm}}} = 1 + 2\frac{\delta\chi^{(3)}}{\chi_0^{(3)}}\xi + \left(\frac{\delta\chi^{(3)}}{\chi_0^{(3)}}\right)^2 \xi. \tag{S10}$$

Here, we consider that:

$$\frac{I_{\text{samp}}}{I_{\text{sub}}} / \frac{\left(\chi_0^{(3)}\right)^2 L^6}{\left(\chi_{sub}^{(3)}\right)^2 L_{sub}^6} = \frac{I_{\text{samp}}^{\text{norm}}}{I_{\text{sub}}^{\text{norm}}}. \tag{S11}$$

Solving Eq. (**??**) gives one Eq. (3) of the main text as a result:

$$\frac{\delta\chi^{(3)}(\tau)}{\chi_0^{(3)}} = \sqrt{\frac{\frac{I_{\text{samp}}^{\text{norm}}(\tau)}{I_{\text{sub}}^{\text{norm}}(\tau)} - 1 + \xi(\tau)}{\xi(\tau)}} - 1. \tag{S12}$$

Generally, expression (3) of the main text is valid for all the delay times $\tau$. However, in the case of large $\tau$, the relative error of the experimental $I_{\text{sub}}$ dependence data is too high to achieve valid results. In the case of $\tau$ is larger than a couple of $\sigma$, we can neglect the coherent effects and deduce the relaxation of $\delta\chi^{(3)}(\tau)$ by measuring the mixed signal $I_{\text{sam}}(f_1+f_2)$ and normalising it over the single-beam signal $I_{\text{sam}}(f_1)$. When the pump pulse has already passed through the metamaterial, from equation (**??**), one can evaluate the following expression:

$$I_{3\omega}(f_1+f_2) \sim 2\chi_0^{(3)}\delta\chi^{(3)}L^6 I_2^3. \tag{S13}$$

Here, for simplicity, we neglected the $\left(\delta\chi^{(3)}/\chi_0^{(3)}\right)^2$ addend; this condition is valid for large time delays. For the case when only one beam interacts with the sample:

$$I_{3\omega}(f_2) \sim \chi_0^{(3)} L^6 I_2^3 \zeta, \tag{S14}$$

where $\zeta$ is the proportionality coefficient between the lock-in signal values detected at $f_2$ and $f_1+f_2$ frequencies. It is extracted from the Fourier transform of the square wave function at the $f_2$ frequency and Fourier transform of the multiplication of square wave functions at the $f_1$ and $f_2$ frequencies. In the case of $f_1 = \frac{6}{5}f_2$, this coefficient equals $\zeta = 2.3$. Thus, one can obtain Eq. (5) of the main text:

$$\frac{\delta\chi^{(3)}}{\chi_0^{(3)}} = \frac{\zeta}{2}\frac{I_{3\omega}(f_1+f_2)}{I_{3\omega}(f_2)}. \tag{S15}$$

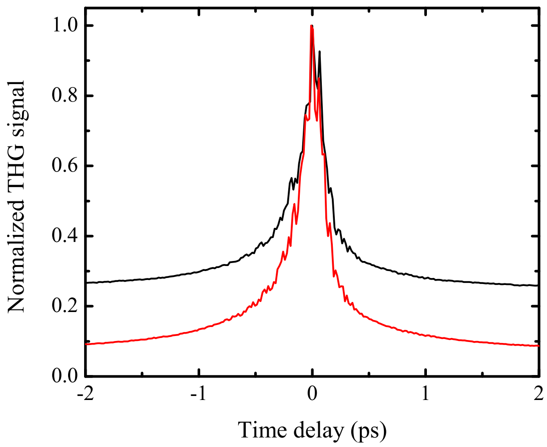


**Figure S1.** Normalized third-harmonic generation signal detected at $f_1$ (black curve) and $f_2$ (red curve) lock-in frequencies.

**Additional experimental data.**
The experimental setup presented in the main text in Fig. 1(d) enables to measure THG signal at both $f_1$ and $f_2$ lock-in frequencies, as well as at $f_1 + f_2$ frequency. The first results for THG time dependence were achieved at $f_1$ and $f_2$ frequencies separately with the aperture removed. These results are presented in Fig. **??**. However it is difficult to extract only mutual effects (e.g. pump pulse excited modulation of the nonlinear susceptibility detected via probe pulse THG modification) from these data because of the offset THG signal generated in each beam in absence of the other beam. In order to avoid it and improve signal-to-noise ratio we detect signal at $f_1 + f_2$ lock-in frequency. These experimental results are presented in Fig. 2 of the main text.
